# Supplementary material for: Mechanisms of RCD-1 pore formation and membrane bending
Source: Nat Commun. 2025 Jan 25;16:1011. doi: 10.1038/s41467-025-56398-5 (PMC11760362; doi:10.1038/s41467-025-56398-5)
Supplement: Supplementary file 1 — Supplementary Information [file 41467_2025_56398_MOESM1_ESM.pdf]

Supplementary Materials

Mechanisms of RCD-1 pore formation and membrane bending

Keli Ren[1], James Daniel Farrell[1,2,3], Yueyue Li[4,5], Xinrui Guo[1,6], Ruipei Xie[1], Xin Liu[6], Qiaozhen Kang[6], Qihui Fan[1], Fangfu Ye[1,7], Jingjin Ding[4,5], Fang Jiao[1]*

[1] Beijing National Laboratory for Condensed Matter Physics, Institute of Physics, Chinese Academy of Sciences, Beijing 100190, PR China.

[2] School of Physical Sciences, University of Chinese Academy of Sciences, Beijing 100049, PR China.

[3] Songshan Lake Materials Laboratory, Dongguan, Guangdong 523808, PR China.

[4] National Laboratory of Biomacromolecules, CAS Center for Excellence in Biomacromolecules, Institute of Biophysics, Chinese Academy of Sciences, Beijing 100101, PR China.

[5] University of Chinese Academy of Sciences, Beijing 101408, PR China.

[6] School of Life Sciences, Zhengzhou University, Zhengzhou 450001, PR China.

[7] Oujiang Laboratory (Zhejiang Lab for Regenerative Medicine, Vision and Brain Health), Wenzhou Institute, University of Chinese Academy of Sciences, Wenzhou, Zhejiang 325000, PR China

*Corresponding to: Fang Jiao (fang.jiao@iphy.ac.cn)

Supplementary Figures

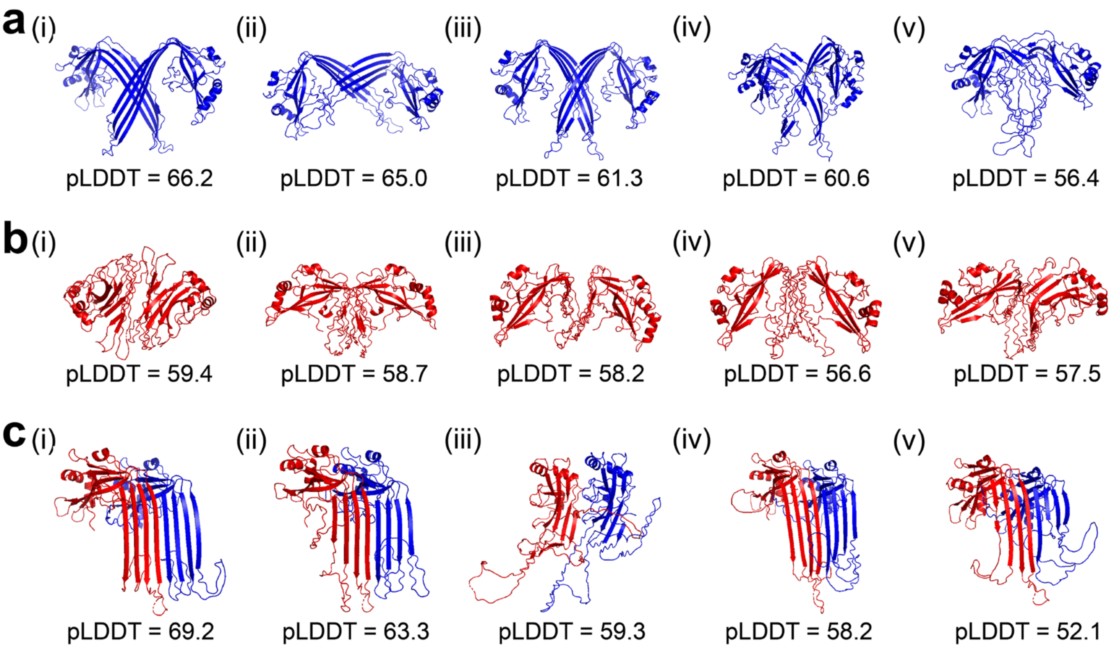

Figure S1. Predicted RCD-1 dimer structures by AlphaFold2-multimer. Predicted structures of (**a**) the RCD-1-1 homodimer, (**b**) the RCD-1-2 homodimer, and (**c**) the RCD-1-1/RCD-1-2 heterodimer. The width of RCD-1-1/RCD-1-2 heterodimer is approximately 5 nm. The five structures with highest pLDDT score are presented in order. RCD-1-1 is colored blue and RCD-1-2 is colored red.

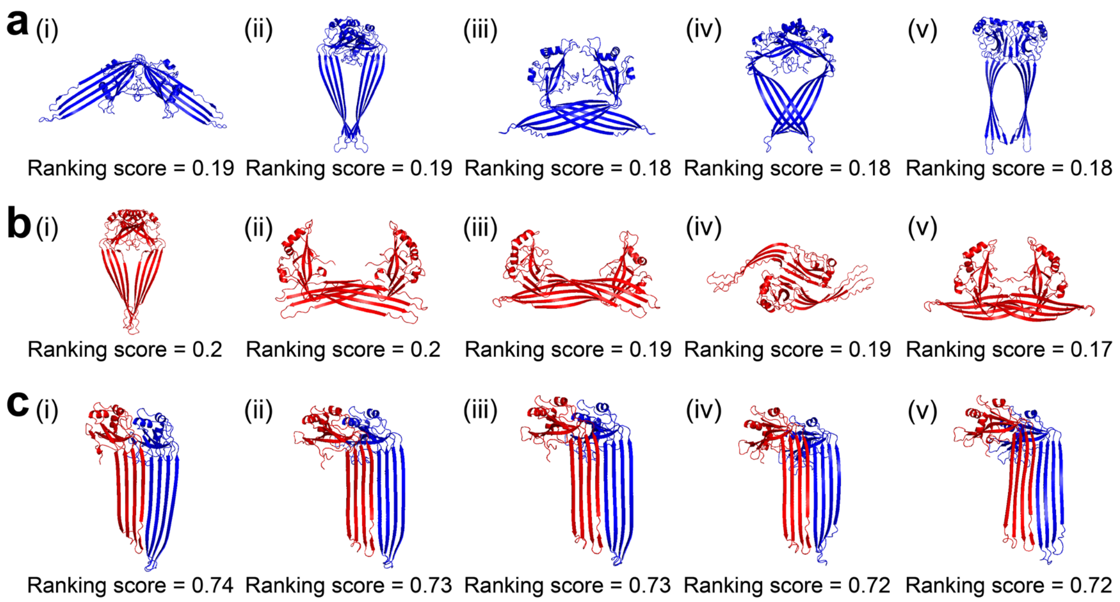

Figure S2. Predicted RCD-1 dimer structures by AlphaFold3. Predicted structures of (**a**) the RCD-1-1 homodimer, (**b**) the RCD-1-2 homodimer, and (**c**) the RCD-1-1/RCD-1-2 heterodimer. The width of RCD-1-1/RCD-1-2 heterodimer is approximately 5 nm. The five structures with highest ranking score are presented in order. RCD-1-1 is colored blue and RCD-1-2 is colored red.

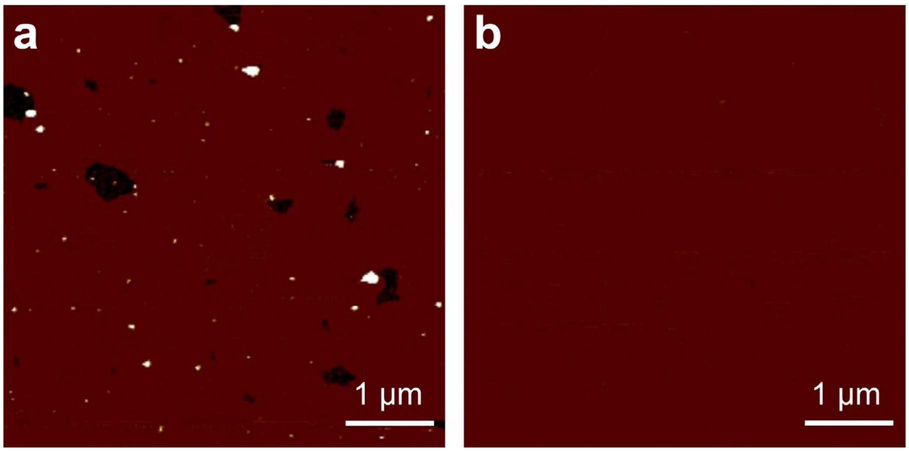

Figure S3. AFM images of SLBs on mica. (**a**) Inadequately prepared SLB (experimental repeats, *n* = 3). The SLB exhibited many defects and impurities, rendering it unsuitable for subsequent protein-related experiments. (**b**) Well prepared SLB (experimental repeats, *n* = 3). The lipid bilayer is defect-free and covers the mica surface uniformly.

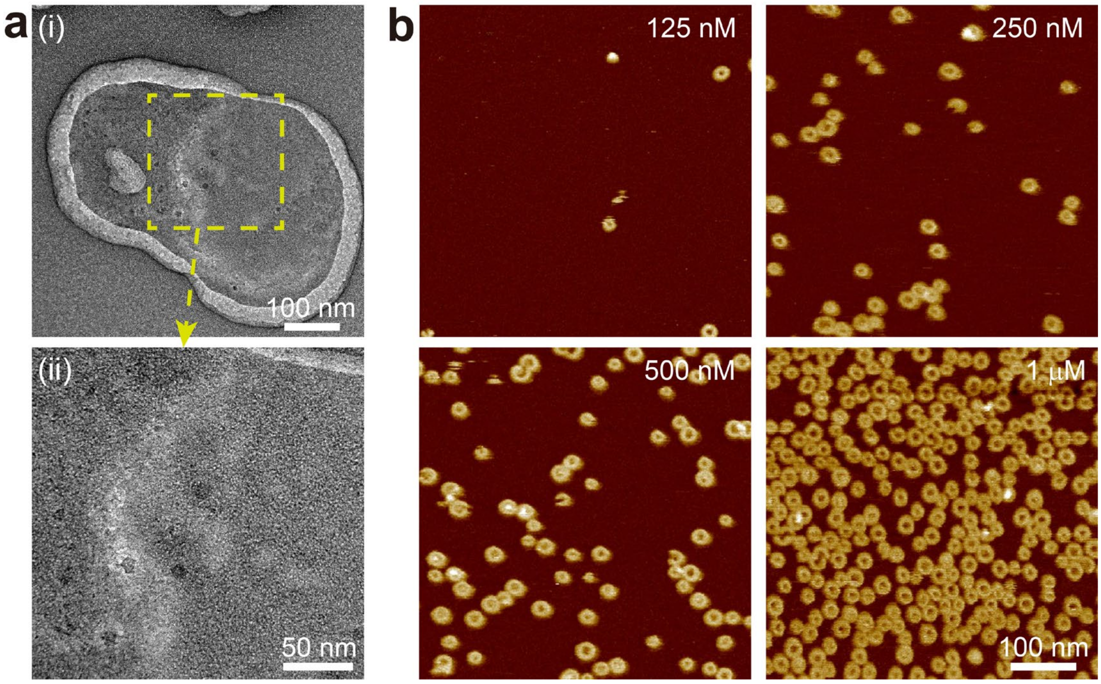

Figure S4. RCD-1 oligomerization on *E. coli* total lipids. (**a**) TEM images of RCD-1 assemblies on RCD-1 in *E. coli* total lipid SLBs (experimental repeats, *n* = 3). (**b**) AFM topographs of RCD-1 co-incubated with *E. coli* total SLBs at concentrations of 125 nM, 250 nM, 500 nM and 1 µM. The number of oligomers increases with protein concentration (experimental repeats, *n* = 3).

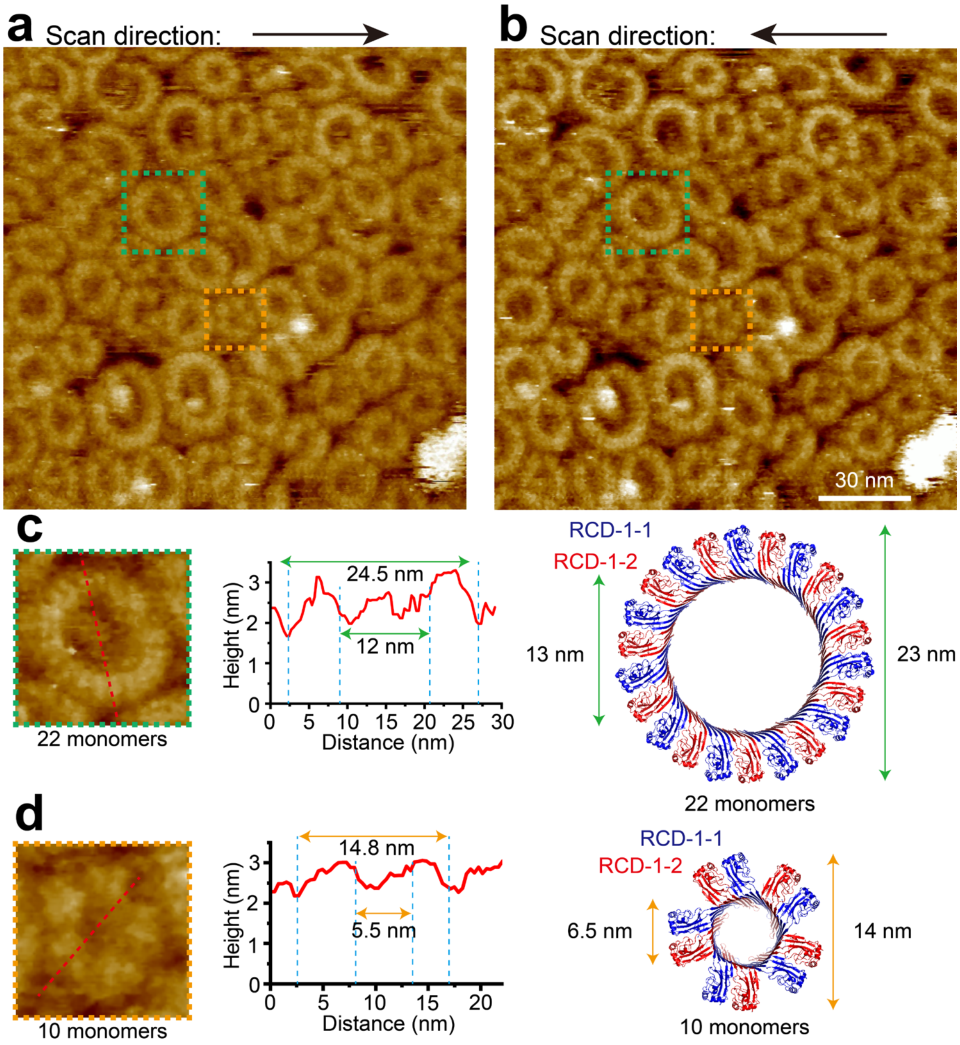

Figure S5. High-resolution AFM images of 1 μM RCD-1 oligomers on SLB made from 6: 4 DOPE: CL (18: 1). Images were consecutively collected from opposite scan directions: (**a**) left to right and (**b**) right to left (**a-b**, experimental repeats, $n = 3$). (**c**) Left, oligomers containing 11 dimers are highlighted within a green square. Middle, the height-section profile measured along the red line indicated in the topography. Right, recently resolved cryo-EM structure of the RCD-1 pore, composed of 11 RCD-1-1/RCD-1-2 heterodimers. (**d**) Left, oligomers containing 5 dimers are highlighted within an orange square. Middle, the height-section profile measured along the red line indicated in the topography. Right, AlphaFold3-predicted RCD-1 pore structure formed by 5 RCD-1-1/RCD-1-2 heterodimers. RCD-1-1 is colored blue and RCD-1-2 is colored red.

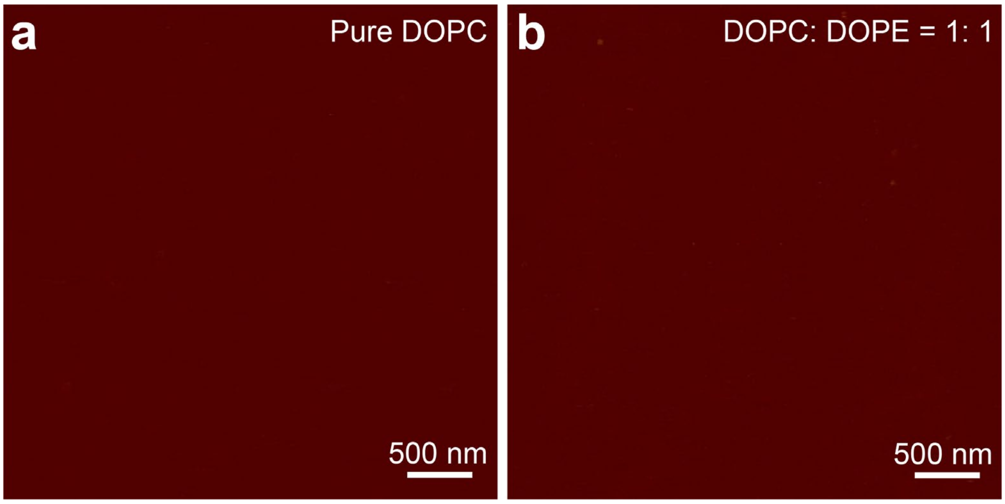

Figure S6. AFM topographies of RCD-1 incubated with SLBs made from PC and PE. AFM topographies of 250 nM RCD-1 incubated with SLBs made from pure PC (**a**) and DOPC: DOPE = 1: 1 (**b**). No oligomers were observed on the SLBs (**a**-**b**, experimental repeats, n = 3).

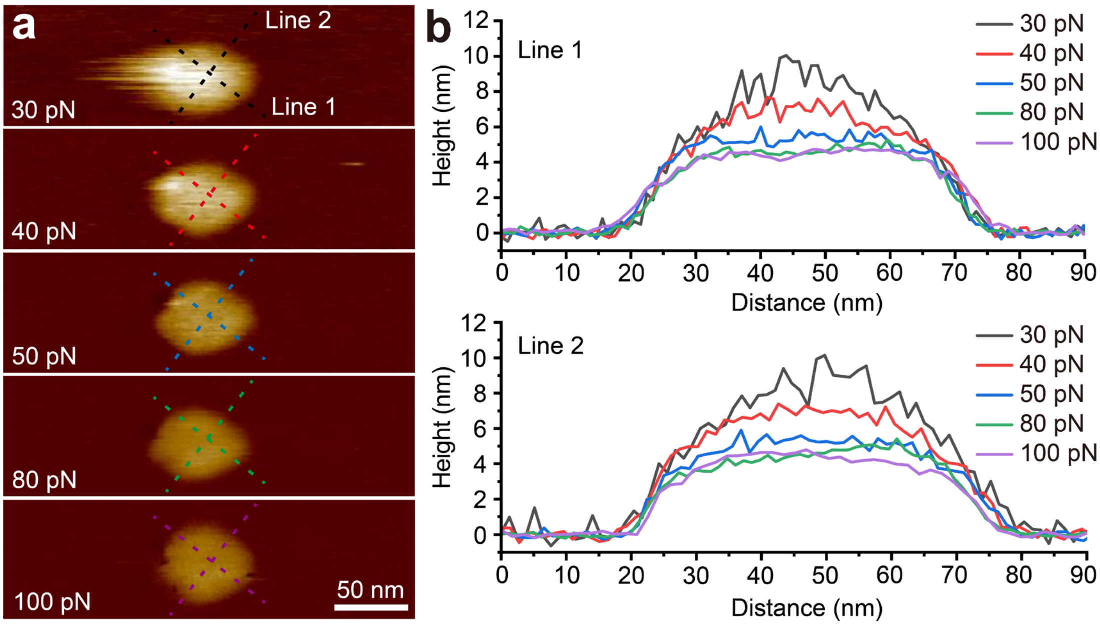

Figure S7. Bent membrane imaged with various forces on an SLB made from DOPC: DOPS = 8: 2. (**a**) AFM images captured with scanning forces of 30, 40, 50, 80, 100 pN (experimental repeats, *n* = 3). (**b**) Height profiles along the dashed lines in (**a**).

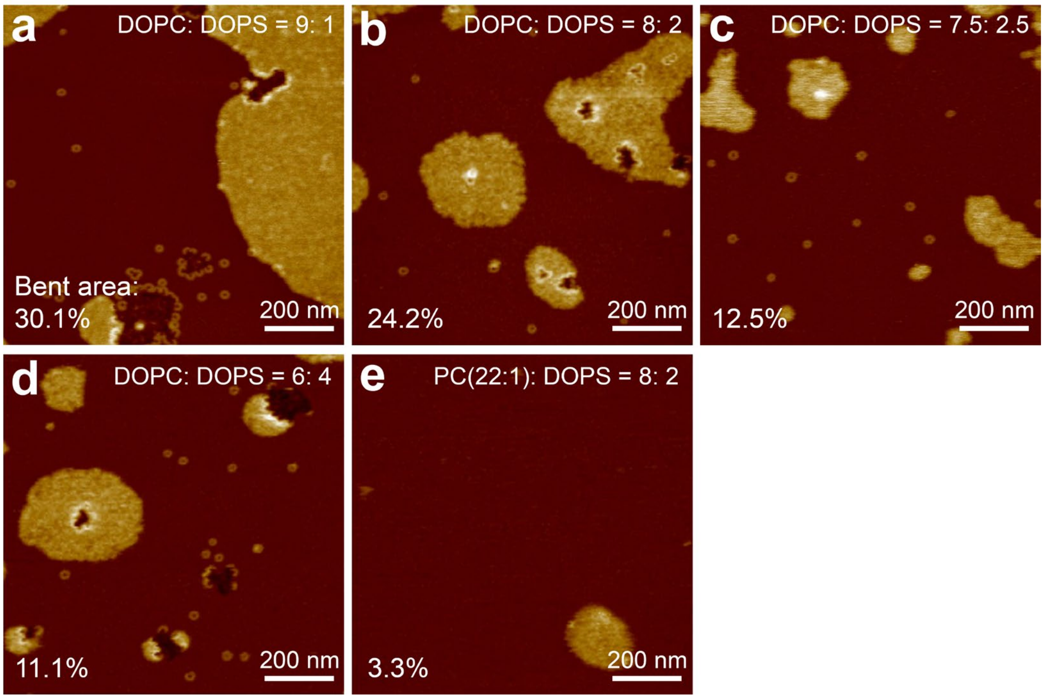

Figure S8. AFM topographies of RCD-1 incubated with SLBs made from PC and PS. AFM topographies of RCD-1 oligomers formed on lipid membranes of (**a**) DOPC: DOPS = 9: 1 (experimental repeats, $n = 3$), (**b**) DOPC: DOPS = 8: 2 (experimental repeats, $n = 10$), (**c**) DOPC: DOPS = 7.5: 2.5 (experimental repeats, $n = 3$), (**d**) DOPC: DOPS = 6: 4 (experimental repeats, $n = 10$), and (**e**) PC (22:1): DOPS = 8: 2 (experimental repeats, $n = 3$). The percentage of bent membrane areas shows 30.1%, 24.2%, 12.5%, 11.1% and 3.3% in conditions of (**a**)-(**e**), respectively.

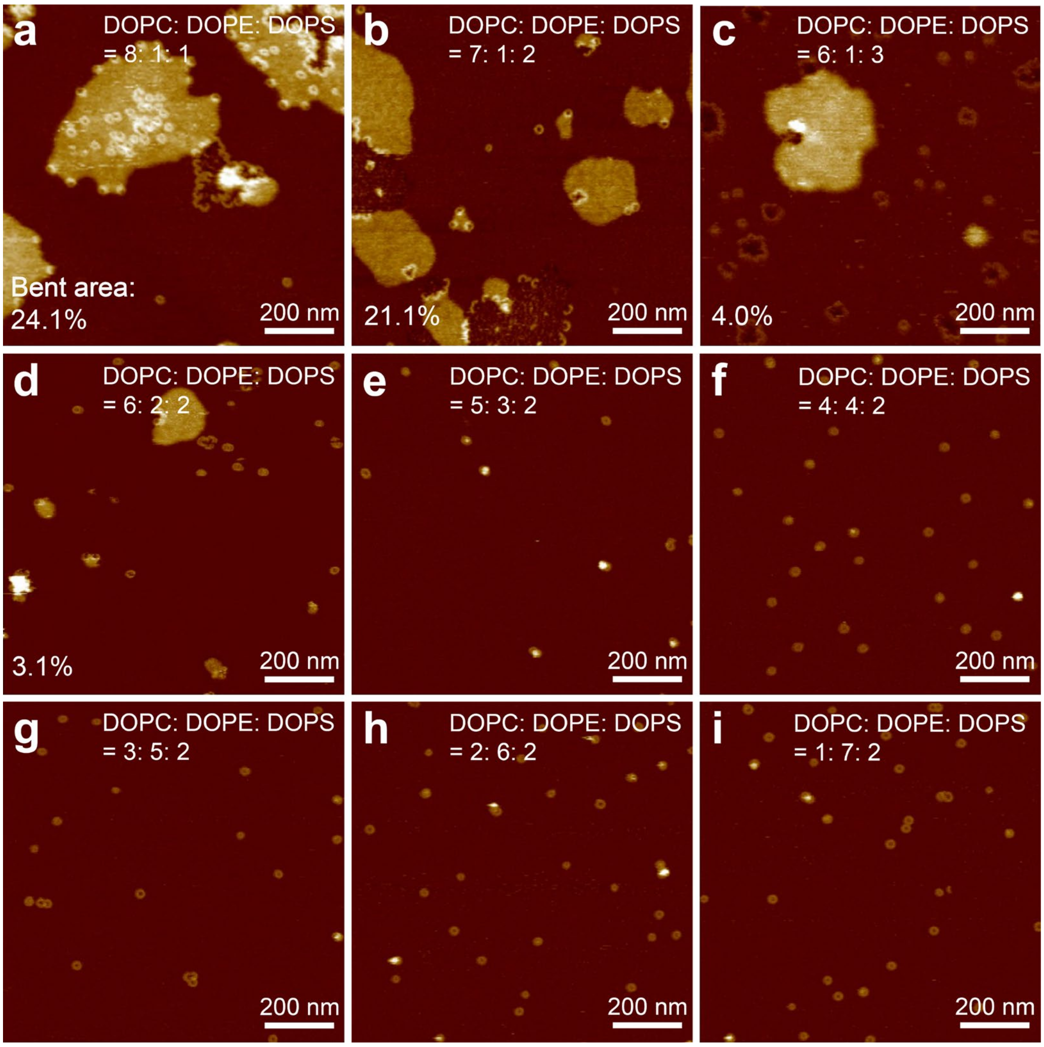

Figure S9. AFM topographies of RCD-1 incubated with SLBs made from PC, PE, and PS. (**a-i**) AFM topographies of RCD-1 oligomers formed on lipid membranes of (**a**) DOPC: DOPE: DOPS = 8: 1: 1, (**b**) DOPC: DOPE: DOPS = 7: 1: 2, (**c**) DOPC: DOPE: DOPS = 6: 1: 3, (**d**) DOPC: DOPE: DOPS = 6: 2: 2, (**e**) DOPC: DOPE: DOPS = 5: 3: 2, (**f**) DOPC: DOPE: DOPS = 4: 4: 2, (**g**) DOPC: DOPE: DOPS = 3: 5: 2, (**h**) DOPC: DOPE: DOPS = 2: 6: 2, and (**i**) DOPC: DOPE: DOPS = 1: 7: 2 (**a-i**, experimental repeats, $n = 3$).

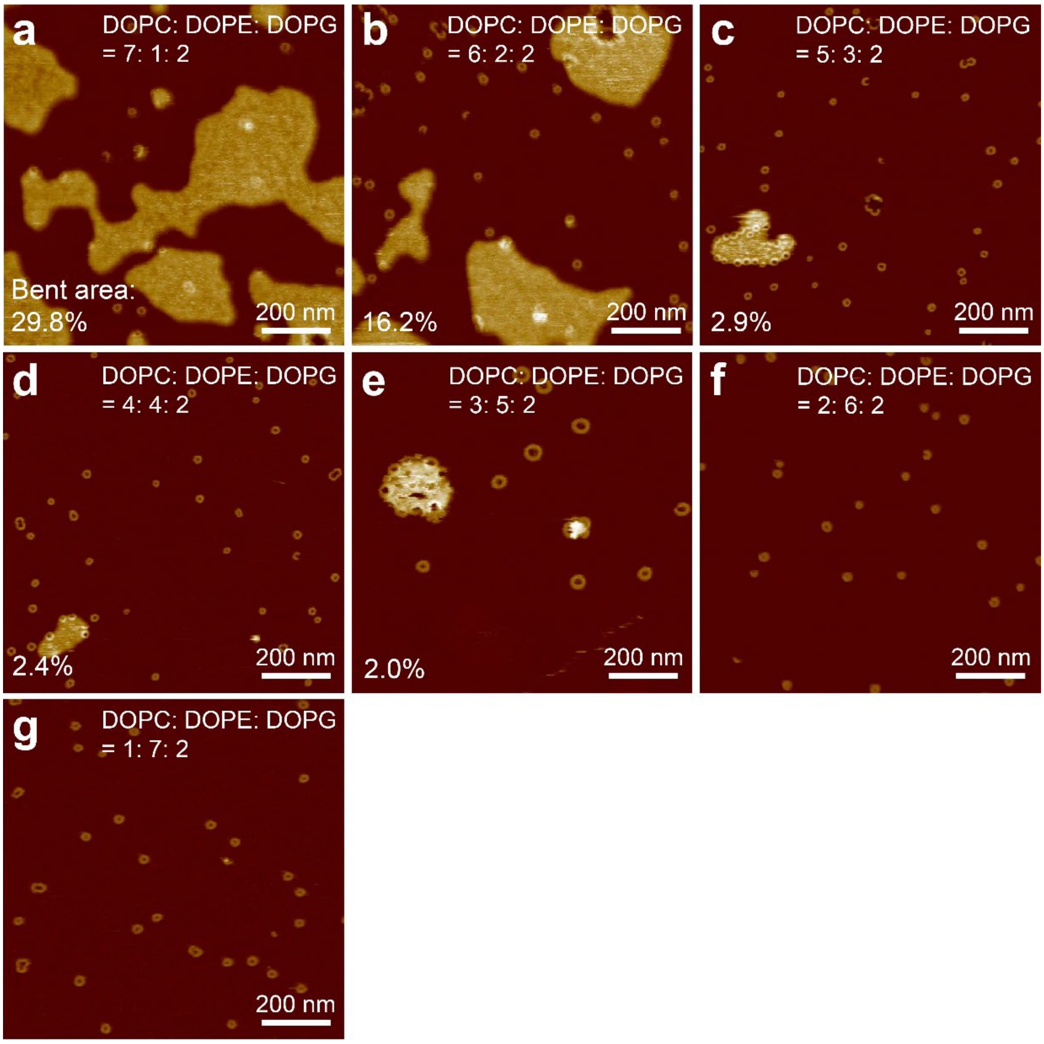

Figure S10. AFM topographies of RCD-1 incubated with SLBs made from PC, PE, and PG. (**a**-**g**) AFM topographies of RCD-1 oligomers formed on lipid membranes of (**a**) DOPC: DOPE: DOPG = 7: 1: 2, (**b**) DOPC: DOPE: DOPG = 6: 2: 2, (**c**) DOPC: DOPE: DOPG = 5: 3: 2, (**d**) DOPC: DOPE: DOPG = 4: 4: 2, (**e**) DOPC: DOPE: DOPG = 3: 5: 2, (**f**) DOPC: DOPE: DOPG = 2: 6: 2, and (**g**) DOPC: DOPE: DOPG = 1: 7: 2 (**a**-**g**, experimental repeats, *n* = 3).

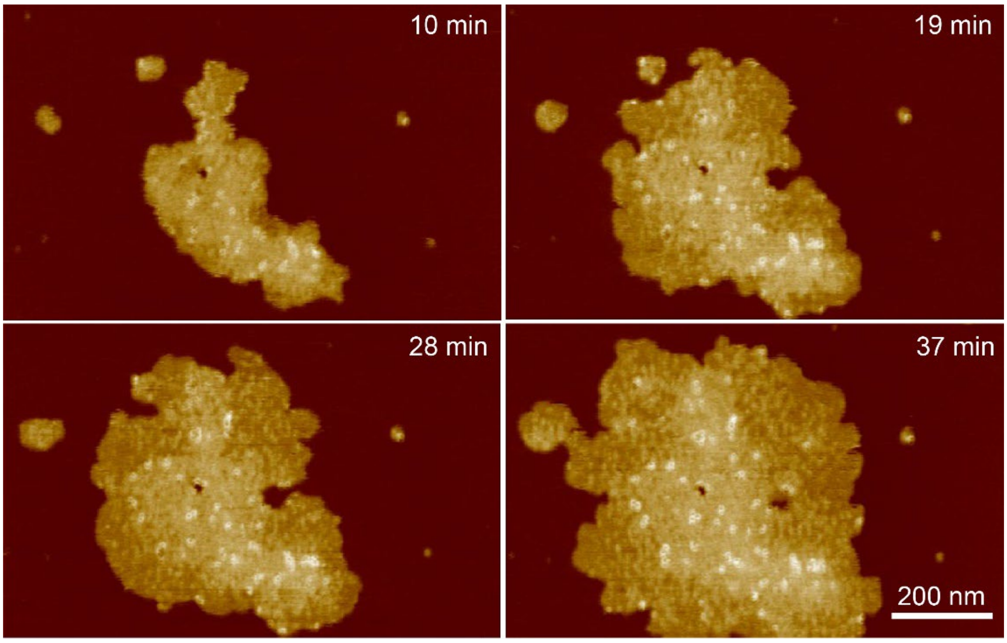

Figure S11. Time-lapse topographies showing small oligomers on the bending area. As the range of membrane bending expands, many small oligomers observed at the new raised membrane surface (experimental repeats, $n = 3$). The SLB is made from DOPC: DOPE: DOPG = 6: 2: 2.

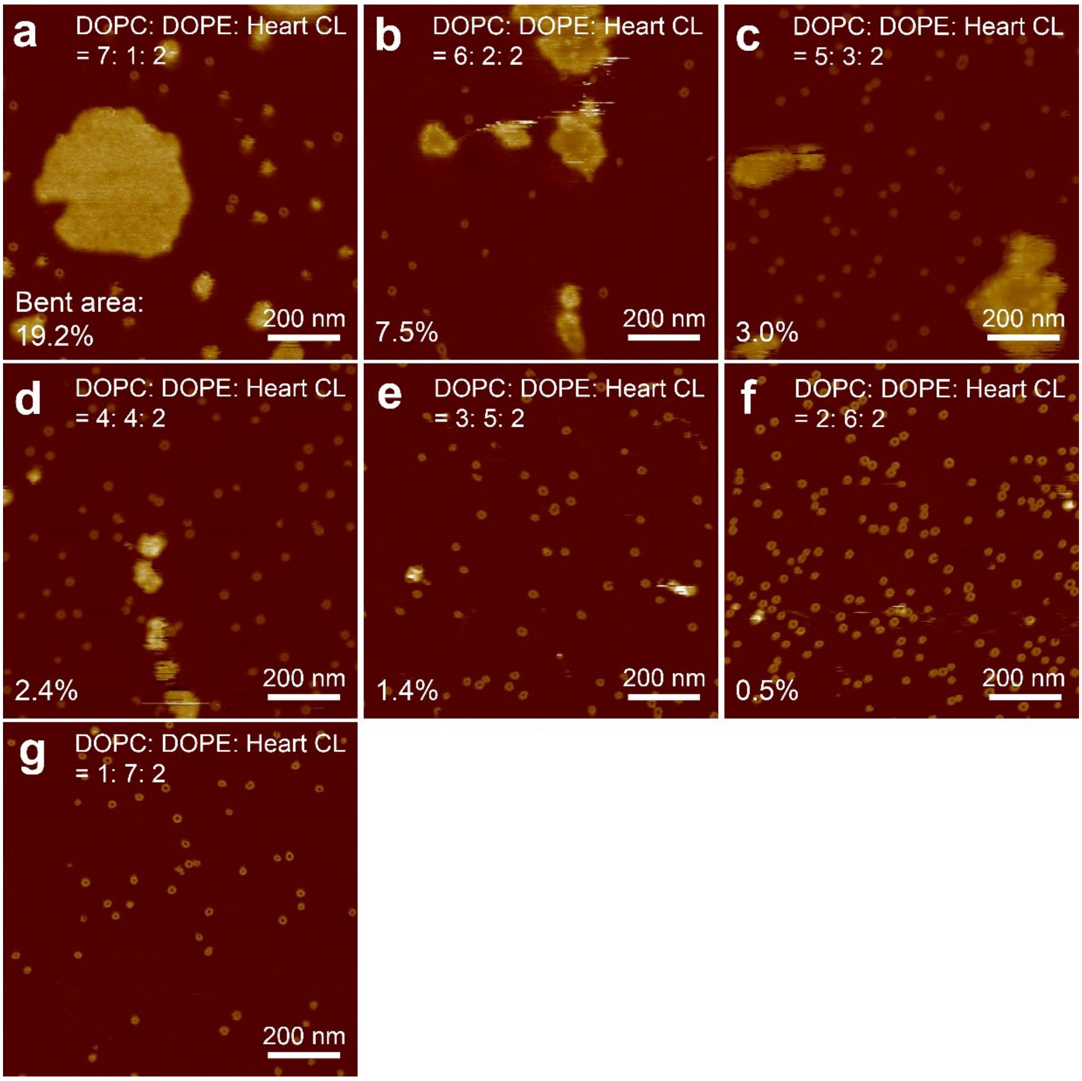

Figure S12. AFM topographies of RCD-1 incubated with SLBs made from PC, PE, and CL. (**a**-**g**) AFM topographies of RCD-1 oligomers formed on lipid membranes of (**a**) DOPC: DOPE: Heart CL = 7: 1: 2, (**b**) DOPC: DOPE: Heart CL = 6: 2: 2, (**c**) DOPC: DOPE: Heart CL = 5: 3: 2, (**d**) DOPC: DOPE: Heart CL = 4: 4: 2, (**e**) DOPC: DOPE: Heart CL = 3: 5: 2, (**f**) DOPC: DOPE: Heart CL = 2: 6: 2, and (**g**) DOPC: DOPE: Heart CL = 1: 7: 2 (**a**-**g**, experimental repeats, n = 3).

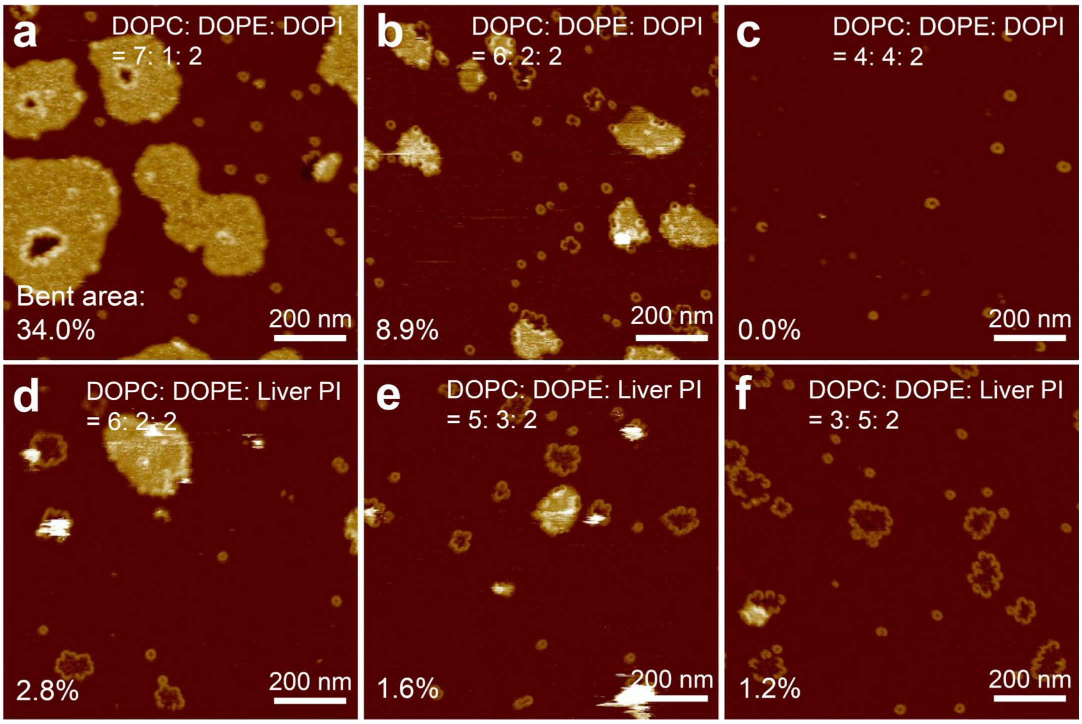

Figure S13. AFM topographies of RCD-1 incubated with SLBs made from PC, PE, and PI. (**a**-**f**) AFM topographies of RCD-1 oligomers formed on lipid membranes of (**a**) DOPC: DOPE: DOPI = 7: 1: 2, (**b**) DOPC: DOPE: DOPI = 6: 2: 2, (**c**) DOPC: DOPE: DOPI = 4: 4: 2, (**d**) DOPC: DOPE: Liver PI = 6: 2: 2, (**e**) DOPC: DOPE: Liver PI = 5: 3: 2, (**f**) DOPC: DOPE: Liver PI = 3: 5: 2 (**a-f**, experimental repeats, $n = 3$).

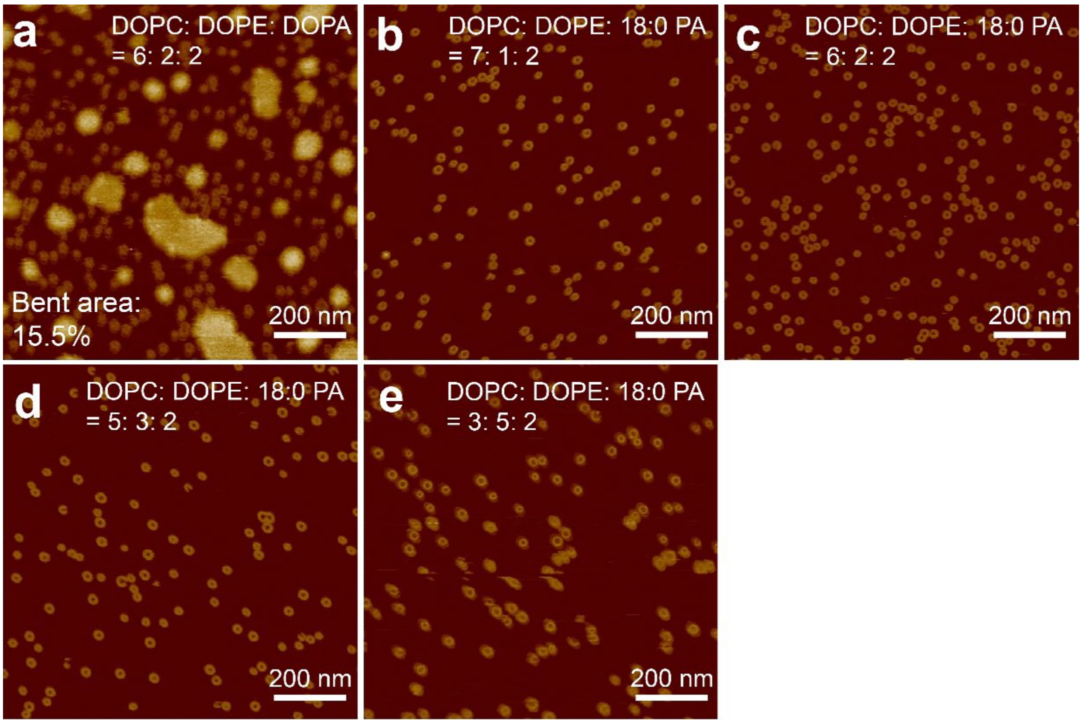

Figure S14. AFM topographies of RCD-1 incubated with SLBs made from PC, PE, and PA. (**a-e**) AFM topographies of RCD-1 oligomers formed on lipid membranes of (**a**) DOPC: DOPE: DOPA = 6: 2: 2, (**b**) DOPC: DOPE: 18: 0 PA = 7: 1: 2, (**c**) DOPC: DOPE: 18: 0 PA = 6: 2: 2, (**d**) DOPC: DOPE: 18: 0 PA = 5: 3: 2, (**e**) DOPC: DOPE: 18: 0 PA = 3: 5: 2 (**a-e**, experimental repeats, *n* = 3).

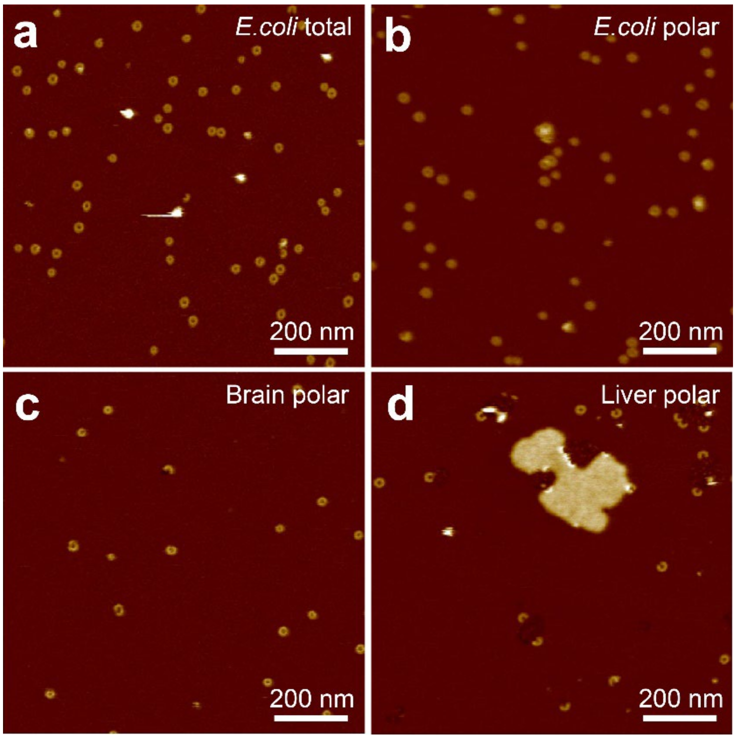

Figure S15. AFM topographies of RCD-1 incubated with SLBs made from natural lipids. (**a**-**d**) AFM topographies of RCD-1 oligomers formed on lipid membranes of (**a**) *E.coli* total, (**b**) *E.coli* polar, (**c**) Brain polar, (**d**) Liver polar (**a**-**d**, experimental repeats, $n = 3$).

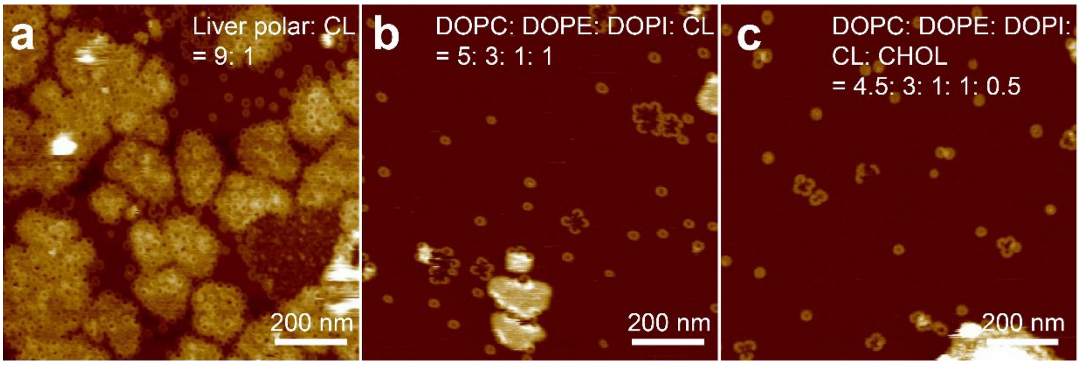

Figure S16. AFM topographies of RCD-1 incubated with SLBs made from mixed lipids. (**a-c**) AFM topographies of RCD-1 oligomers formed on lipid membranes of (**a**) Liver polar: Heart CL = 9: 1, (**b**) DOPC: DOPE: DOPI: Heart CL = 5: 3: 1: 1, (**c**) DOPC: DOPE: DOPI: Heart CL: cholesterol = 4.5: 3: 1: 1: 0.5 (**a-c**, experimental repeats, *n* = 3).

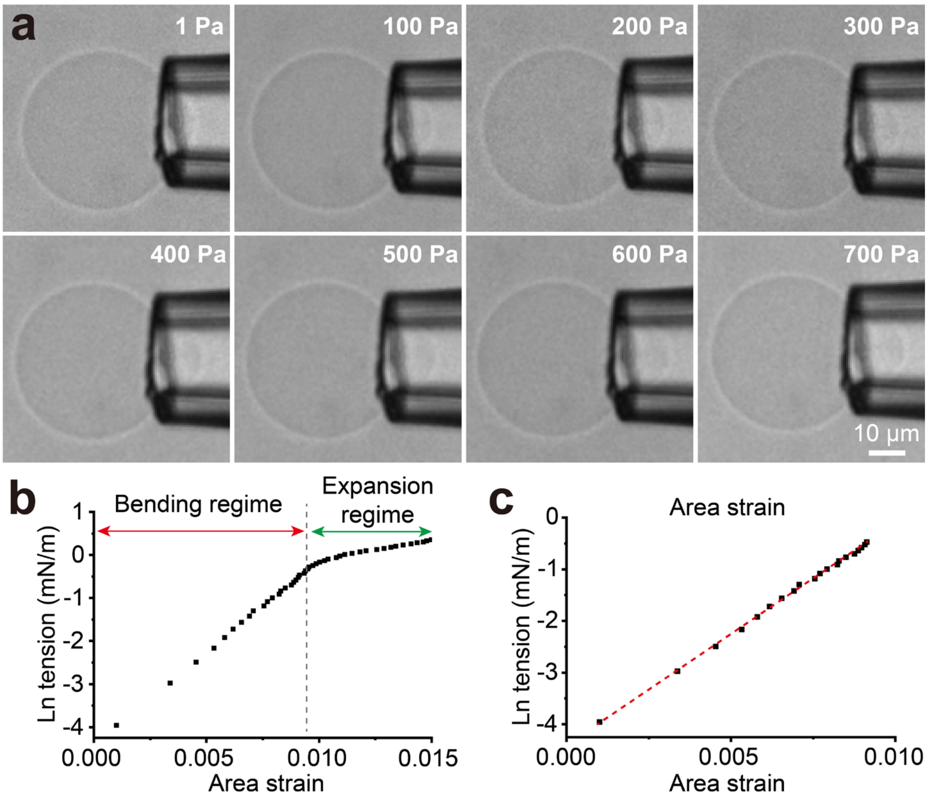

Figure S17. Micropipette aspiration measurements of the bending modulus of GUVs composed of DOPC: DOPS = 8: 2. (**a**) Bright field images showing changes in GUV morphology with increasing suction pressure (experimental repeats, n = 10). (**b**) Tension–strain measurements for the GUV. (**c**) Linear fit of the low-tension regime in panel (b), where the slope (dashed lines) yielding the elastic bending moduli k_c (× 8π/k_BT).

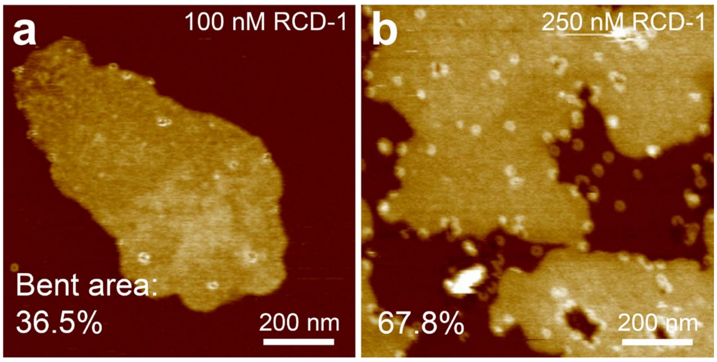

Figure S18. AFM topographies of RCD-1 incubated with yeast extract polar membranes. AFM images show the incubation of (**a**) 100 nM RCD-1 and (**b**) 250 nM RCD-1 on yeast extract polar membranes, with bent membrane areas covering 36.5% and 67.8% in (**a**) and (**b**), respectively (**a-b**, experimental repeats, n = 3).

Supplementary Table 1

RCD-1 oligomerization in different SLBs

Lipid Compositions	Membrane Bending	Bending Area (%)	Incubation Concentration	Incubation Time
**PC PS**				
DOPC: DOPS = 9: 1	**Yes**	30.1	250nM	5mins
DOPC: DOPS = 8: 2	**Yes**	24.2	250nM	5mins
DOPC: DOPS = 7.5: 2.5	**Yes**	12.5	250nM	5mins
DOPC: DOPS = 6: 4	**Yes**	11.1	250nM	5mins
PC(22:1): DOPS = 8: 2	**Yes**	3.3	250nM	5mins
**PC PE PS**				
DOPC: DOPE: DOPS = 8: 1: 1	**Yes**	24.1	250nM	5mins
DOPC: DOPE: DOPS = 7: 1: 2	**Yes**	21.1	250nM	5mins
DOPC: DOPE: DOPS = 6: 1: 3	**Yes**	4.0	250nM	5mins
DOPC: DOPE: DOPS = 6: 2: 2	**Yes**	3.1	250nM	5mins
DOPC: DOPE: DOPS = 5: 3: 2	No		250nM	5mins
DOPC: DOPE: DOPS = 4: 4: 2	No		250nM	5mins
DOPC: DOPE: DOPS = 3:5:2	No		250nM	5mins
DOPC: DOPE: DOPS = 2:6:2	No		250nM	5mins
DOPC: DOPE: DOPS = 1:7:2	No		250nM	5mins
**PC PE PG**				
DOPC: DOPE:DOPG = 7: 1: 2	**Yes**	29.8	250nM	5mins
DOPC: DOPE:DOPG = 6: 2: 2	**Yes**	16.2	250nM	5mins
DOPC: DOPE:DOPG = 5: 3: 2	**Yes**	2.9	250nM	5mins
DOPC: DOPE:DOPG = 4: 4: 2	**Yes**	2.4	250nM	5mins
DOPC: DOPE:DOPG = 3: 5: 2	**Yes**	2.0	250nM	5mins
DOPC: DOPE:DOPG = 2: 6: 2	No		250nM	5mins
DOPC: DOPE:DOPG = 1: 7: 2	No		250nM	5mins
**PC PE CL**				
DOPC: DOPE: Heart CL = 7: 1: 2	**Yes**	19.2	250nM	5mins
DOPC: DOPE: Heart CL = 6: 2: 2	**Yes**	7.5	250nM	5mins
DOPC: DOPE: Heart CL = 5: 3: 2	**Yes**	3.0	250nM	5mins
DOPC: DOPE: Heart CL = 4: 4: 2	**Yes**	2.4	250nM	5mins
DOPC: DOPE: Heart CL = 3: 5: 2	**Yes**	1.4	250nM	5mins

DOPC: DOPE: Heart CL = 2: 6: 2	**Yes**	0.5	250nM	5mins
DOPC: DOPE: Heart CL = 1: 7: 2	No		250nM	5mins
**PE CL**				
DOPE: CL(18:1) = 6: 4	No		1μM	5mins
**PC PE PI**				
DOPC: DOPE: DOPI = 7: 1: 2	**Yes**	34.0	250nM	5mins
DOPC: DOPE: DOPI = 6: 2: 2	**Yes**	8.9	250nM	5mins
DOPC: DOPE: DOPI = 4: 4: 2	No		250nM	5mins
DOPC: DOPE: Liver PI = 6: 2: 2	**Yes**	2.8	500nM	20mins
DOPC: DOPE: Liver PI = 5: 3: 2	**Yes**	1.6	500nM	20mins
DOPC: DOPE: Liver PI = 3 :5: 2	**Yes**	1.2	500nM	20mins
**PC PE PA**				
DOPC: DOPE: DOPA = 6: 2: 2	**Yes**	15.5	250nM	5mins
DOPC: DOPE: PA(18:0) = 7: 1: 2	No		250nM	5mins
DOPC: DOPE: PA18:0 = 6:2:2	No		250nM	5mins
DOPC: DOPE: PA(18:0) = 5:3:2	No		250nM	5mins
DOPC: DOPE: PA(18:0) = 3:5:2	No		250nM	5mins
**Natural Lipids**				
E.coli Total	No		125nM	5mins
E.coli Total	No		250nM	5mins
E.coli Total	No		500nM	5mins
E.coli Total	No		1μM	5mins
E.coli Polar	No		250nM	5mins
Liver Polar	**Yes**	2.8	250nM	5mins
Brain Polar	No		250nM	5mins
Yeast Extract Polar	**Yes**	36.5	100nM	5mins
Yeast Extract Polar	**Yes**	67.8	250nM	5mins
**Mixed Lipids**				
Liver Polar: Heart CL = 9: 1	**Yes**	68.8	1000nM	40mins
DOPC: DOPE: Liver PI: Heart CL=5: 3: 1: 1	**Yes**	3.9	250nM	5mins
DOPC: DOPE: Liver PI: Heart CL: Cholesterol = 4.5: 3: 1: 1: 0.5	**Yes**	2.8	250nM	5mins
**Artificial Fungal Lipids**				
**Artificial Fungal Cell Membrane**				

DOPC: DOPE: DOPA: DOPS: CL(18:1) = 30: 28: 20: 4: 18	**Yes**	82.1	250nM	5mins
**Modified Artificial Fungal Mitochondrial Membrane**				
DOPC: DOPE: PA(18:0): DOPS: CL(18:1) = 30: 28: 20: 4: 18	No		250nM	5mins
**Artificial Fungal Mitochondrial Membrane**				
DOPC: DOPE: DOPA: DOPI: DOPS: CL(18:1): DOPG = 37: 43: 1: 8: 4: 6: 1	No		250nM	5mins
**Artificial Fungal Mitochondrial Outer Membrane**				
DOPC: DOPE: DOPA: DOPI: DOPS: CL(18:1):DOPG = 40: 36: 4: 9: 5: 5: 1	No		250nM	5mins
**Artificial Fungal Mitochondrial Inner Membrane**				
DOPC: DOPE: DOPA: DOPI: DOPS: CL(18:1): DOPG = 32: 30: 1: 5: 5: 25: 2	**Yes**	1.2	250nM	5mins
**Modified Artificial Fungal Mitochondrial Inner Membrane**				
DOPC: DOPE: DOPA: DOPI: DOPS: Heart CL(18:2): DOPG = 32: 30: 1: 5: 5: 25: 2	**Yes**	7.4	250nM	5mins
