# Supplementary material for: Mechanisms of RCD-1 pore formation and membrane bending
Source: Nat Commun. 2025 Jan 25;16:1011. doi: 10.1038/s41467-025-56398-5 (PMC11760362; doi:10.1038/s41467-025-56398-5)
Supplement: Supplementary file 2 — Reporting Summary [file 41467_2025_56398_MOESM2_ESM.pdf]

nature portfolio

Corresponding author(s): Fang Jiao

Last updated by author(s): Dec 19, 2024

Reporting Summary

Nature Portfolio wishes to improve the reproducibility of the work that we publish. This form provides structure for consistency and transparency in reporting. For further information on Nature Portfolio policies, see our Editorial Policies and the Editorial Policy Checklist.

Statistics

For all statistical analyses, confirm that the following items are present in the figure legend, table legend, main text, or Methods section.

n/a	Confirmed	
☐	☒	The exact sample size (*n*) for each experimental group/condition, given as a discrete number and unit of measurement
☐	☒	A statement on whether measurements were taken from distinct samples or whether the same sample was measured repeatedly
☒	☐	The statistical test(s) used AND whether they are one- or two-sided<br>*Only common tests should be described solely by name; describe more complex techniques in the Methods section.*
☒	☐	A description of all covariates tested
☒	☐	A description of any assumptions or corrections, such as tests of normality and adjustment for multiple comparisons
☐	☒	A full description of the statistical parameters including central tendency (e.g. means) or other basic estimates (e.g. regression coefficient) AND variation (e.g. standard deviation) or associated estimates of uncertainty (e.g. confidence intervals)
☒	☐	For null hypothesis testing, the test statistic (e.g. *F*, *t*, *r*) with confidence intervals, effect sizes, degrees of freedom and *P* value noted<br>*Give P values as exact values whenever suitable.*
☒	☐	For Bayesian analysis, information on the choice of priors and Markov chain Monte Carlo settings
☒	☐	For hierarchical and complex designs, identification of the appropriate level for tests and full reporting of outcomes
☒	☐	Estimates of effect sizes (e.g. Cohen's *d*, Pearson's *r*), indicating how they were calculated

Our web collection on statistics for biologists contains articles on many of the points above.

Software and code

Policy information about availability of computer code

Data collection	AFM (Multimode 8, Bruker); Confocal fluorescence microscope (FV3000, Olympus); Negative Stain TEM(JEOL2100, JEOL); Micropipette aspiration was controlled by a micromanipulator (InjectMan 4, Eppendorf) and the images were captured by an inverted phase contrast microscope (Nikon Ti-E).
Data analysis	AFM data were processed using NanoScope Analysis 3.00 (Bruker); GUVs images were processed with Fiji (ImageJ); Micropipette aspiration images were processed with Fiji (ImageJ)

For manuscripts utilizing custom algorithms or software that are central to the research but not yet described in published literature, software must be made available to editors and reviewers. We strongly encourage code deposition in a community repository (e.g. GitHub). See the Nature Portfolio guidelines for submitting code & software for further information.

Data

Policy information about availability of data

All manuscripts must include a data availability statement. This statement should provide the following information, where applicable:

- Accession codes, unique identifiers, or web links for publicly available datasets
- A description of any restrictions on data availability
- For clinical datasets or third party data, please ensure that the statement adheres to our policy

The values from all bar graphs displayed in this manuscript as well as uncropped scans of all AFM images are supplied in the Source Data file. The cryo-EM structure of RCD-1 pore comprised by 11 dimers can be accessed by PDB under accession number 8JYZ [https://doi.org/10.2210/pdb8JYZ/pdb].

Research involving human participants, their data, or biological material

Policy information about studies with human participants or human data. See also policy information about sex, gender (identity/presentation), and sexual orientation and race, ethnicity and racism.

Reporting on sex and gender	N/A
Reporting on race, ethnicity, or other socially relevant groupings	N/A
Population characteristics	N/A
Recruitment	N/A
Ethics oversight	N/A

Note that full information on the approval of the study protocol must also be provided in the manuscript.

Field-specific reporting

Please select the one below that is the best fit for your research. If you are not sure, read the appropriate sections before making your selection.

☒ Life sciences ☐ Behavioural & social sciences ☐ Ecological, evolutionary & environmental sciences

For a reference copy of the document with all sections, see nature.com/documents/nr-reporting-summary-flat.pdf

Life sciences study design

All studies must disclose on these points even when the disclosure is negative.

Sample size	No statistical methods were applied to pre-determine sample sizes and no sample size calculation was performed.
Data exclusions	No data were excluded from the analysis.
Replication	Each replicable experiment was independently repeated at least three times. The number of replicates performed for each figure is stated in the figure legends. AFM experiments were successfully repeated across different samples, days and AFM tips. Biochemical experiments, including protein purification, were conducted successfully in biological replicates for more than 10 times
Randomization	There was no randomization for these experiments. This study is not a randomised control trial and randomisation is not conventionally used in in vitro/in cellulo studies such as this one. All groups of experiments were performed using the same experimental conditions and protocols
Blinding	Assays used objective quantification methods that are not susceptible to bias, so samples were not blinded

Reporting for specific materials, systems and methods

We require information from authors about some types of materials, experimental systems and methods used in many studies. Here, indicate whether each material, system or method listed is relevant to your study. If you are not sure if a list item applies to your research, read the appropriate section before selecting a response.

Materials & experimental systems

n/a	Involved in the study
☒	☐ Antibodies
☒	☐ Eukaryotic cell lines
☒	☐ Palaeontology and archaeology
☒	☐ Animals and other organisms
☒	☐ Clinical data
☒	☐ Dual use research of concern
☒	☐ Plants

Methods

n/a	Involved in the study
☒	☐ ChIP-seq
☒	☐ Flow cytometry
☒	☐ MRI-based neuroimaging

Plants

Seed stocks

N/A

Novel plant genotypes

N/A

Authentication

N/A
